# Differential effects of fentanyl compared to morphine on neuroinflammatory signaling in the brain in EcoHIV-infected mice

**DOI:** 10.1007/s13365-025-01252-z

**Published:** 2025-05-30

**Authors:** Kara Rademeyer, Austin M. Jones, Emily A. Miller, Daniel Conway, Joseph L. McClay, Kurt F. Hauser, MaryPeace McRae

**Affiliations:** 1 Department of Pharmacotherapy and Outcomes Science, Virginia Commonwealth University, Richmond, VA 23298 U.S.A.; 2 Department of Biomedical Engineering, The Ohio State University, Columbus, OH 43210 U.S.A.; 3 Department of Pharmacology and Toxicology, School of Medicine, Virginia Commonwealth University, Richmond, VA U.S.A.; 4 Department of Anatomy and Neurobiology, School of Medicine, Virginia Commonwealth University, Richmond, VA U.S.A.

**Keywords:** Fentanyl, Morphine, EcoHIV, Inflammation, Opioid abuse, Blood–brain barrier, Chemokines

## Abstract

HIV-associated neurocognitive disorders (HAND) pose significant challenges, particularly in individuals with comorbid opioid use disorder (OUD). Fentanyl, a potent synthetic opioid, is a leading contributor to opioid-related fatalities, yet its effects in the context of HIV remain poorly understood. This study investigated the differential impacts of fentanyl and morphine on neuroinflammatory signaling, blood–brain barrier (BBB) integrity, and antiretroviral (ARV) brain accumulation in EcoHIV-infected mice. C57BL/6 mice were assigned to morphine, fentanyl, or saline treatment groups, with or without EcoHIV infection. Tight junction proteins claudin-5 and ZO-1 were measured in striatum and hippocampus via ELISA, while proinflammatory chemokines were analyzed by multiplex assay. In EcoHIV-infected mice, both opioids significantly increased CCL2, CCL4, CCL5, and CCL11 concentrations in the striatum, with fentanyl causing greater increases in CCL2 than morphine. Additionally, fentanyl, but not morphine, significantly decreased CCL3 concentrations in the striatum. Principal component analysis revealed distinct treatment-specific patterns within the striatum, underscoring opioid-specific differences. Tight junction protein expression data demonstrate opioid-specific, sex-specific, and region-specific effects on the key BBB proteins, ZO-1 and claudin-5. Both opioids also reduced ARV brain concentrations in infected mice, with region- and opioid-specific effects. Fentanyl decreased dolutegravir in the hippocampus, while morphine decreased abacavir in both regions. These findings demonstrate that fentanyl and morphine exposure in the context of HIV produce distinct impacts on neuroinflammatory response, BBB integrity, and ARV brain exposure. Understanding these opioid-specific effects is critical for improving clinical outcomes and treatment strategies for individuals with HIV and OUD.

**Supplementary Information:**

The online version contains supplementary material available at 10.1007/s13365-025-01252-z.

## Introduction

Of the estimated 1.2 million people living with HIV in the United States, up to 8% also struggle with comorbid opioid use disorder (OUD) and these individuals experience poorer health outcomes than either condition alone. In addition to the established health risks of opioid use in general, opioids pose specific risks within the context of HIV infection (Salters et al. 2021; Aden et al. 2015; Cachay et al. 2023; Rosen et al. 2013; Jeevanjee et al. 2014). For example, several studies in both humans and animal models indicate that substance use can exacerbate the deleterious consequences of neuro-acquired human immunodeficiency virus (neuroHIV) infection, which can result in specific cognitive deficits—collectively referred to as HIV-associated neurocognitive disorders (HAND) (Bell et al. 1998; Byrd et al. 2013, 2011; Nath et al. 2000; Anthony et al. 2008; Meyer et al. 2013; Fitting et al. 2020; Wallace 2022; Waki and Freed 2010; Chilunda et al. 2019). In addition, chronic opioid exposure within the context of HIV infection is associated with upregulated neuroinflammation and deficits in neuronal function with possible injury, and can also lower the concentrations of some antiretroviral (ARV) drugs in the brain (Verani et al. 2005; Olivier et al. 2018; Osborne et al. 2020; Leibrand et al. 2019; Fantuzzi et al. 2003; Letendre et al. 2004; El-Hage et al. 2005; Martin et al. 2018; Kruize and Kootstra 2019).

Chronic opioid exposure uniquely interacts with HIV pathology, particularly in the brain, although the mechanisms are not fully established. Fentanyl, a highly potent, synthetic opioid µ-opioid receptor (MOR) agonist, has quickly become one of the most abused drugs in the US. In 2015, 29% of all opioid-related overdose deaths were attributed to fentanyl, but by 2021, this proportion had increased to 89% (Comer and Cahill 2019; National Safety Council 2024). Most of what we know about the effects of long-term opioid use on neuroHIV comes from studies of morphine and heroin, which also target the MOR. Due to this common mechanism of action, researchers often contend that their long-term effects are likely to be similar. However, fentanyl is approximately 50–400 times more potent than morphine and 30–50 times more potent than intravenous heroin depending on the bioassay (Comer and Cahill 2019; Moss and Carlo 2019; Ciccarone et al. 2017). Additionally, fentanyl exhibits a vastly different pharmacological profile, acting as an agonist at both MOR and other opioid and non-opioid receptors, whereas morphine and heroin have few actions at non-opioid receptors (Torralva and Janowsky 2019; Torralva et al. 2020; Kelly et al. 2023). The dramatic differences in potency and pharmacological properties (e.g., receptor specificity) are relevant because they impact degree of receptor activation and the resulting downstream effects, leading to heightened neuroinflammatory and neurotoxic processes associated with HIV. Therefore, we hypothesize that fentanyl impacts the brain uniquely from morphinan opioids such as heroin and morphine, and likely to poses unique risks for people living with HIV (PLWH).

To our knowledge, there are few studies examining the effects of fentanyl in neuroHIV. Although several studies have looked at the impact of chronic morphine use on neuroinflammatory signaling, antiretroviral CNS concentrations, and blood–brain barrier (BBB) integrity in PLWH, very few have studied fentanyl. Our lab recently reported that fentanyl caused profound disruption to BBB integrity (i.e., tight junction protein expression, leakage analyses) and changes to neuroimmune signaling in HIV-1 Tat transgenic mice (Rademeyer et al. 2024). In other prior work using this same model, we found that chronic morphine exposure also changes the pattern of neuroinflammatory signaling, expression of BBB proteins, and the accumulation of ARV drugs (Leibrand et al. 2019, 2022). To the extent that preclinical findings in mice can be generalized to humans, all three effects have the potential to significantly impact clinical outcomes for PWLH. Though these studies suggest there may be overlap in the ways fentanyl and morphine impact neuropathology within the context of HIV, no study, to date, has included a direct comparison of the two drugs.

In this study, we directly compare the effects of morphine vs fentanyl using an infectious EcoHIV mouse model. In the EcoHIV model, there is an initial burst of viral replication that is limited by an adaptive immune response (Kelschenbach et al. 2012). Despite the suppression of virus, EcoHIV infection invades the brain (He et al. 2014; Potash et al. 2005; Kim et al. 2019), causes BBB dysfunction (Jones et al. 2016), results in increased systemic as well as brain cytokine responses (Potash et al. 2005; Sindberg et al. 2015) and deficits in working memory, spatial learning and contextual fear memory as early as 21 days post infection (Gu et al. 2018). Building on our prior work in HIV-1 Tat-expressing transgenic mice, we explored the effects of opioids in the infectious EcoHIV mouse model on neuroinflammatory signaling (i.e., chemokine expression), BBB integrity (i.e., tight junction protein expression), and antiretroviral accumulation in the brain in mice. We found that fentanyl, like morphine, reduces ARV accumulation and decreases the expression of tight junction proteins. However, our data indicate that fentanyl has a unique impact on the pattern of chemokine expression in the brain, particularly in the striatum where neuroinflammation is likely to affect motor and reward systems, including key aspects of cognitive function (e.g., motivation, saliency, and planning). These findings suggest an opioid-specific approach is necessary to fully appreciate and address the impact of drug use on clinical outcomes among PLWH.

## Materials and methods

### Animal subjects

Male and female C57BL/6 mice (Envigo, ~ 4 months of age weighing ~ 25 g) were used in these studies. For all experiments, we established a total of six treatment groups, where each group consisted of *n = *8 mice (4 males/4 females). Two groups received morphine treatment, and two received fentanyl (+/− EcoHIV infection). All studies were approved by the Institutional Animal Care and Use Committee at Virginia Commonwealth University, and the experiments were conducted in accordance with ethical guidelines defined by the National Institutes of Health (National Research Council (US) Committee for the Update of the Guide for the Care and Use of Laboratory Animals 2011).

### EcoHIV propagation and infection in mice

EcoHIV is constructed by genetic engineering replacing the gp120 HIV glycoprotein with the gp80 from ecotropic murine leukemia virus, which only infects rodents (Potash et al. 2005). EcoHIV mimics active viral infection. Virus can be detected in the brain, spleen, and peritoneal macrophages within 2–12 weeks after a single inoculation (He et al. 2014; Potash et al. 2005; Gu et al. 2018). EcoHIV infection elicits similar responses as HIV, such as increased chemokine secretion within the brain, neurotoxicity, and impaired cognition (Dong et al. 2020; Kelschenbach et al. 2012; Olson et al. 2018). To establish the model, EcoHIV stocks were prepared through the transfection of 293 T cells by plasmid EcoHIV DNA, as described previously (Potash et al. 2005). The resultant EcoHIV was titered for core antigen, p24 content (Gu et al. 2018). Male and female C57BL/6 mice were anesthetized (3–4% isoflurane) and injected intraperitoneally with 2 × 10^6^ pg of p24 EcoHIV [EcoHIV(+)] or with normal sterile saline [EcoHIV(−), uninfected mice]. Mice were housed 4 per cage and were separated by infection status. Food and water were provided ad libitum for the duration of the study.

### Antiretroviral and opioid drug treatments

Triumeq® tablets, purchased from the VCU Health Systems Pharmacy, were used to prepare a drug solution formulated to deliver abacavir (2.5 mg/day), lamivudine (1.2 mg/day), and dolutegravir (0.2 mg/day). Doses were prepared using allometric scaling as previously described (Nair and Jacob 2016). This antiretroviral regimen was chosen for its clinical relevance as a recommended single-tablet, daily HIV treatment and to ensure consistency with our prior studies on morphine’s impact on antiretroviral concentrations in Tat transgenic mice (Panel on Antiretroviral Guidelines for Adults and Adolescents 2024). Fentanyl (NIDA Drug Supply Program, Bethesda, MD), morphine (NIDA Drug Supply Program, Bethesda, MD) or sterile saline (controls) was added to the ARV solution and loaded into the Alzet® pump at a concentration sufficient to deliver 1.92 mg/day morphine, 0.05 mg/day fentanyl, or saline. ARVs were co-administered with each treatment (morphine, fentanyl, or saline) for 5 days. Drug preparations were made in batches to minimize dosing variability.

### Surgical manipulation

Fifteen days post inoculation, Alzet® osmotic pumps (2001, 1.0 μL/h, Cupertino, CA) containing ARV drugs ± morphine, fentanyl, or saline were subcutaneously implanted in the mice. After 5 days of continuous delivery of ARVs ± opioids, mice were anesthetized (4% isoflurane) and sacrificed by exsanguination to collect blood. The brain and spleen were harvested from each mouse and ½ of fresh spleen was used for DNA isolation for PCR verification of infection by qPCR, as previously described (Hadas et al. 2007). All the mice in these studies received ARVs. Therefore, the impact of ARVs on brain EcoHIV replication were not assessed as part of the experimental design. The brain was hemisected into left and right hemispheres, and further microdissected into striatum and hippocampus. Tissues were snap frozen and stored at − 80 °C until analysis. Plasma was separated from whole blood by centrifugation (5,000 × *g* at 4 °C for 10 min) and stored at − 80 °C until analysis.

### Antiretroviral quantification analysis

ARV concentrations were measured in the plasma and tissues using LC–MS/MS, as described previously (Leibrand et al. 2019). Briefly, the right striatum and hippocampus frozen samples were weighed and homogenized using Precellys® hard tissue grinding kit tubes (Cayman Chemical, MI, USA) containing 1 mL of 70:30 acetonitrile:1 mM ammonium phosphate (pH 7.4). Lamivudine and abacavir plasma and tissue homogenates were extracted by protein precipitation with the isotopically labeled internal standards, abacavir-d 4 and lamivudine-15 N-d 2. Chromatographic separation was achieved by reverse phase chromatography on a Waters Atlantis T3 (50 × 2.1 mm, 3 µm) analytical column. Dolutegravir plasma and tissue homogenates were extracted by protein precipitation with the isotopically labeled internal standard, dolutegravir-13 C-d 5. Chromatographic separation was achieved by reverse phase chromatography on a Waters XTERRA MS C18 (50 × 2.1 mm, 3.5 μm) analytical column. An AB Sciex API-5000 triple quadrupole mass spectrometer was used to detect all the analytes and internal standards under positive ion electrospray conditions. Precision and accuracy of the calibration standards and quality control samples were within 15% (lamivudine, abacavir in plasma) or 20% (lamivudine, abacavir in tissues and dolutegravir in plasma and tissues) for the following dynamic ranges: 50.0—4,000 ng/mL of lamivudine in plasma; 5.00—4,000 ng/mL of abacavir in plasma; 20.0—4,000 ng/mL of dolutegravir in plasma; 0.100—50.0 ng/mL of lamivudine, abacavir in tissue; and 0.025—50.0 ng/mL of dolutegravir in tissue. Final concentrations were normalized to tissue mass and reported in ng/g units.

### Enzyme-linked immunosorbent assay

Enzyme-linked Immunosorbent Assay (ELISA) was performed on left striatal and hippocampal tissue lysates to determine whether EcoHIV and morphine or fentanyl exposure alters the concentrations of claudin-5 and ZO-1 in the striatum and hippocampus. Presence and relative amounts of tight junction and tight junction accessory protein in striatal and hippocampal samples were detected using the mouse tight junction protein 1 (ZO1) ELISA kit (#MBS2604345, MyBioSource, San Diego, CA) and mouse claudin 5 (CLDN5) ELISA kit per manufacturer’s instructions (#MBS456204, MyBioSource, San Diego, CA). Briefly, 5 µg samples of striatal and hippocampus lysates from the left hemisphere were used. For ZO-1, samples were incubated in pre-coated microplate wells, followed by washing steps to remove unbound antibodies and impurities. Biotinylated antibodies that are specific for ZO-1 or claudin-5 were added to each well, then horseradish + avidin, and finally substrates for the horseradish peroxidase (HRP) reaction. The microplate was washed thoroughly before each step. The microplates were then read on a BioTek plate reader using an absorbance of 450 nm (Synergy H1, 269,978, BioTek). During the plate reading process, samples were normalized to the blank-control wells that contained only the standard diluent for ZO-1 or diluent buffer for claudin-5. Sample concentrations were extrapolated from a standard curve. Samples were run in duplicate and the averages were used for statistical analysis.

### Chemokine assay

Chemokines were measured in the left striatum and left hippocampus using a Mouse 13-plex Proinflammatory Chemokine Panel, (Catalog #740,007; BioLegend) according to the manufacturer’s instructions. The striatal and hippocampal tissues were homogenized separately using NP40 lysis buffer containing cOmplete™ Mini Protease Inhibitor Cocktail (Roche Diagnostics, Germany). The homogenates were centrifuged (16,000 × *g* at 4 °C for 10 min) and the extracted supernatants were used to determine protein content using the Pierce™ BCA Protein Assay Kit (Thermo Fisher Scientific) and then stored in − 80 °C. For chemokine analysis, samples were prepared according to the manufacturer (BioLegend) and were analyzed by the Cytek® Aurora flow cytometer (Cytek® Biosciences, Fremont, California). Sample lysates containing 12.5 μg of protein or standard were loaded in duplicate and both sample and standard wells were incubated with chemokine antibody-conjugated beads (12.5 μL) for 2 h, followed by incubation with biotinylated detection antibodies for 1 h. During both incubation periods, the filter plate was placed on a shaker at 500 rpm. After both incubations, streptavidin-PE (SA-PE) was added for signal amplification and one final incubation period of 30 min was conducted. The filter plate was washed and sealed and stored at 4 °C for 24 h until analysis. Immediately before analysis, samples were washed and resuspended for 5 min, and then transferred to a u-bottom plate. The concentration of each chemokine was determined using five-parameter logistic regression analysis (LEGENDplex™ V8.0 Data Analysis Software Suite (BioLegend)) against known standards.

## Statistical analyses

### Measuring the effects on tight junction proteins Claudin-5 and ZO-1

Sample lysates used for ELISA analysis for claudin-5 and ZO-1 were performed in duplicate. Duplicates were averaged and fit to a standard curve to determine claudin-5 and ZO-1 concentrations for the females and males in the striatum and hippocampus. The resultant concentrations of claudin-5 and ZO-1 in females and males within the striatum and hippocampus were used to conduct a two-way analysis of variance (two-way ANOVA) to determine significant main effects and interactions between infection status and treatment (fentanyl, morphine, or placebo) in the striatum and hippocampus. The Sidak’s multiple comparison test was used for pairwise comparisons between group means.

### Measuring the effects on ARV concentration and chemokine expression

To analyze the effects of EcoHIV infection and/or opioid treatment (fentanyl, morphine, or saline) on ARV and chemokine concentrations, two-way analyses of variance (two-way ANOVA) were performed on averaged, duplicate samples of ARVs and chemokines in the striatum and hippocampus. Sidak’s multiple comparison test was used to make pairwise comparisons between group means. When determining ARV concentrations in tissues, plasma-normalized values that were below the lower limit of quantification (LLOQ) were set to zero [Plasma LLOQ: abacavir 5 ng/mL, lamivudine 50 ng/mL, dolutegravir 20 ng/mL; Tissue LLOQ dependent on sample]. For chemokine analyses, the data was collapsed across the sexes, and the chemokine results were analyzed as described above. The chemokines, CCL20, CXCL1, CXCL5, and CXCL9 were below the limit of detection and therefore not included in the statistical analysis. Statistical analyses were performed, and results graphed using GraphPad Prism 9.0, v9.4.2 (GraphPad Software, LLC, La Jolla, CA). A significance level of α = 0.05 was used and if the calculated *p*-value was less than *p < *0.05 results were considered statistically significant. Uninfected mice are represented as EcoHIV(–) or Eco(–).

### Principal component analysis

To explore chemokine expression patterns among treatment groups, we also assessed chemokine concentrations within the striatum and hippocampus using multivariate methods (Helmy et al. 2012). A principal component analysis (PCA) was conducted on the striatum and hippocampus tissue concentrations of the chemokines that were above the limit of quantification to compare effects among each intervention: EcoHIV status, opioid exposure, and sex. Concentrations for each chemokine were centered and scaled by subtracting the mean and dividing by the standard deviation. Number of retained principal components (PCs) were selected by determining which PCs described most of the variance within the data through scree plot visualization. The chemokines (loadings) that contributed the most to each retained PC were described. One outlier within the striatum data (a male, morphine treated mouse) was removed from the analysis.

### Hierarchical clustering

Hierarchical clustering was also performed on striatum and hippocampus chemokine levels to understand if patterns arose when EcoHIV status, opioid exposure, and sex were assessed simultaneously. Euclidean distance and complete linkage clustering methods best represented the fitted data. Chemokine data was scaled as described above. The outlier described previously was also excluded from the hierarchical clustering analysis.

## Results

### Evidence of a difference in the pattern of changes to chemokine concentrations in response to fentanyl vs morphine in brain tissue from mice infected with EcoHIV

#### Differences in the pattern of changes to chemokine concentrations

**Fig. 1:**
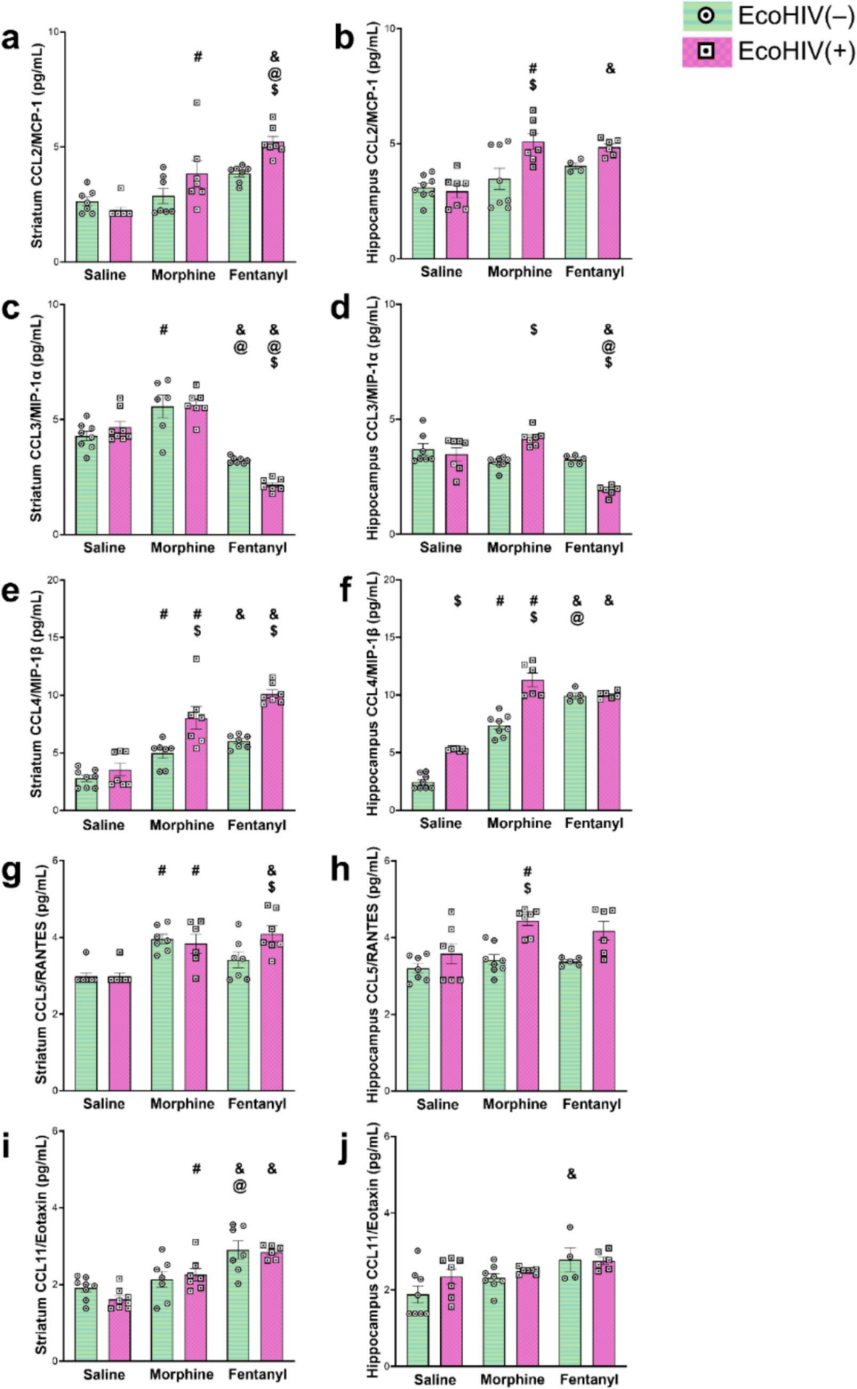
Effects of morphine, fentanyl, and EcoHIV on inflammatory chemokines in the striatum and hippocampus. EcoHIV(–) mice are depicted by horizontal green lines and white circles with a black dot. EcoHIV(+) mice are depicted by pink squares and white squares with a black dot. Data represent the mean concentration for each chemokine ± SEM, sampled from *n = *4–8 per experimental group. Sidak’s multiple comparison post-hoc analysis was used to account for multiple testing. Significant differences at α < 0.05 are denoted by ^#^*p < *0.05 saline vs. morphine, ^&^*p < *0.05 saline vs. fentanyl, ^@^*p < *0.05 morphine vs. fentanyl, or ^$^*p < *0.05 EcoHIV(–) vs. EcoHIV(+) for post-hoc testing

**Fig. 2:**
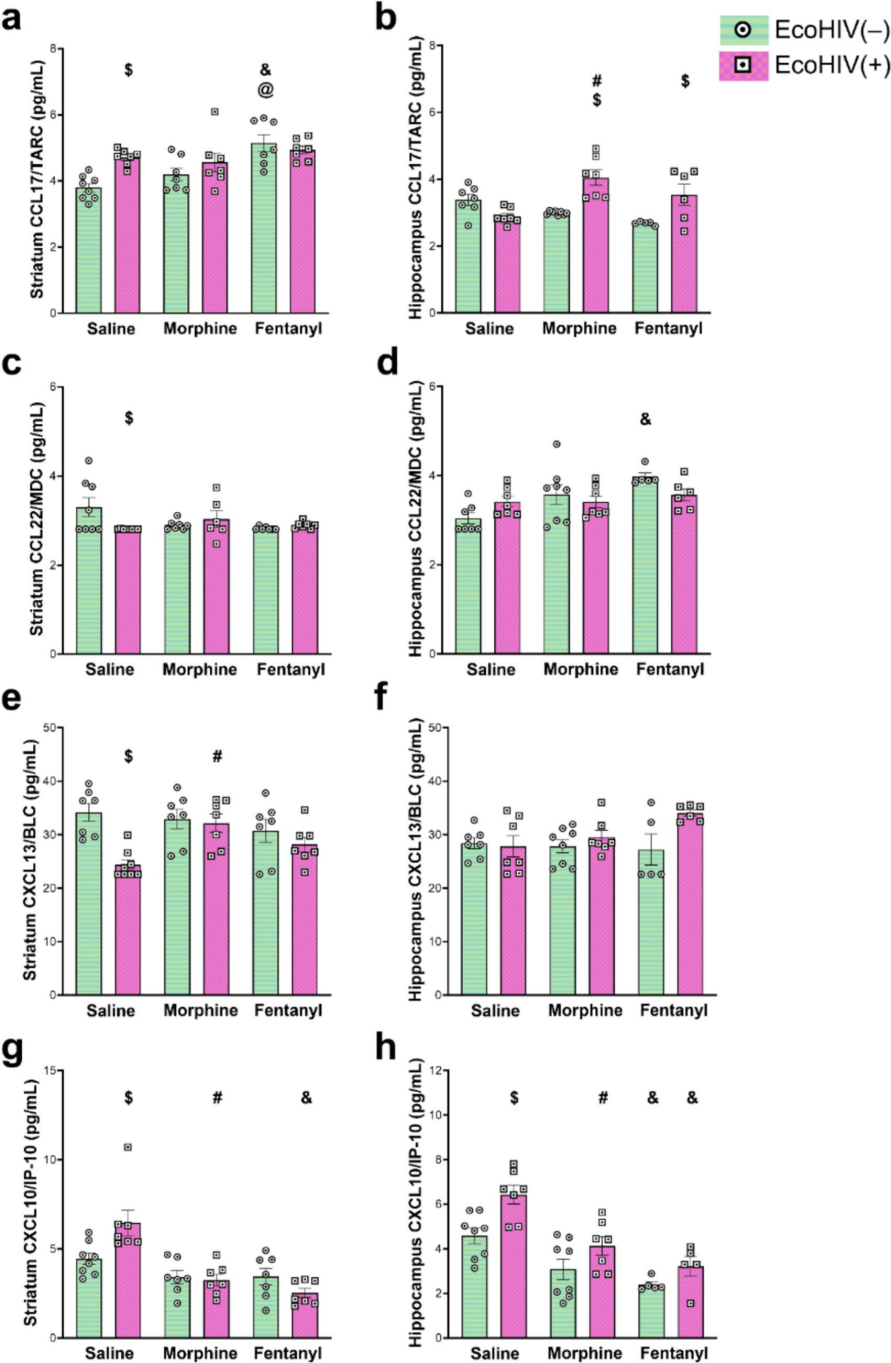
Effects of morphine, fentanyl, and EcoHIV on dual-function and homeostatic chemokines in the striatum and hippocampus. EcoHIV(–) mice are depicted by horizontal green lines and white circles with a black dot. EcoHIV(+) mice are depicted by pink squares and white squares with a black dot. Data represent the mean concentration for each chemokine ± SEM, sampled from *n = *4–8 per experimental group. Sidak’s multiple comparison post-hoc analysis was used to account for multiple testing. Significant differences at α < 0.05 are denoted by ^#^*p < *0.05 saline vs. morphine, ^&^*p < *0.05 saline vs. fentanyl, ^@^*p < *0.05 morphine vs. fentanyl, or ^$^*p < *0.05 EcoHIV(–) vs. EcoHIV(+) for post-hoc testing

Several studies have reported that morphine exposure impacts the concentration of various chemokines in the brain (Gonek et al. 2018; Campbell et al. 2013; Turchan-Cholewo et al. 2009; Mahajan et al. 2002). In prior work, we found that chronic fentanyl exposure dysregulates chemokine signaling in transgenic HIV-1 Tat mice. In this current study, we also find that fentanyl exposure changes chemokine signaling in EcoHIV-infected wild type mice (Figs. 1, 2, Tables S2, S3). Statistical details, including the *F* values and degrees of freedom, are provided in Table 1.

**Table 1:** Effects of fentanyl or morphine and EcoHIV exposure on chemokine levels in the striatum and hippocampus

Brain Region		Opioid/Treatment (Main effect)		EcoHIV (Main Effect)		Interaction	
**Striatum**							
	Chemokine	*F*_(2,37)_	*p*	*F*_(1,37)_	*p*	*F*_(2,37)_	*p*
	CCL2	24.36	** < 0.0001**⟣	7.01	**0.01**⫚	4.794	**0.01**⩷
	CCL3	68.71	** < 0.0001**⟣	1.025	0.31	5.117	**0.01**⩷
	CCL4	46.34	** < 0.0001**⟣	38.30	** < 0.0001**⫚	5.517	**0.008**⩷
	CCL5	19.49	** < 0.0001**⟣	2.054	0.16	3.510	**0.04**⩷
	CCL11	25.66	** < 0.0001**⟣	0.3268	0.57	0.9692	0.38
	CCL17	9.741	**0.0004**⟣	5.260	**0.02**⫚	4.398	**0.01**⩷
	CCL22	1.416	0.255	1.009	0.32	4.417	**0.01**⩷
	CXCL10	19.33	** < 0.0001**⟣	0.7878	0.38	6.327	**0.004**⩷
	CXCL13	2.512	0.09	10.92	**0.002**⫚	4.381	**0.01**⩷
**Hippocampus**							
	Chemokine	*F*_(2,34)_	*p*	*F*_(1,34)_	*p*	*F*_(2,34)_	*p*
	CCL2	12.05	**0.0001**⟣	7.889	**0.008**⫚	4.247	**0.02**⩷
	CCL3	19.42	** < 0.0001**⟣	1.153	0.29	21.28	** < 0.0001**⩷
	CCL4	249.5	** < 0.0001**⟣	80.70	** < 0.0001**⫚	18.95	** < 0.0001**⩷
	CCL5	5.176	**0.01**⟣	24.82	** < 0.0001**⫚	1.811	0.1789
	CCL11	6.558	**0.003**⟣	2.089	0.15	0.9634	0.39
	CCL17	3.773	**0.03**⟣	11.27	**0.002**⫚	12.59	** < 0.0001**⩷
	CCL22	5.675	**0.007**⟣	0.2387	0.62	3.243	0.05
	CXCL10	22.90	** < 0.0001**⟣	13.46	**0.0008**⫚	0.8908	0.41
	CXCL13	1.347	0.27	4.422	**0.04**⫚	2.729	0.07

Main effects are represented by significant main effect of treatment (⟣) [morphine or fentanyl vs. saline-treated], significant main effect of EcoHIV status (⫚) [EcoHIV(−) vs. EcoHIV(+)], and the interaction between Treatment and EcoHIV status (⩷)

To test our hypothesis that fentanyl and morphine have distinct effects on neuroinflammatory signaling within the context of HIV, we tracked changes in the concentration of nine chemokines—CCL2/MCP-1, CCL3/MIP-1α, CCL4/MIP-1β, CCL5/RANTES, CCL11/eotaxin, CCL17, CCL22, CXCL13 and CXCL10—in response to fentanyl or morphine exposure (five-day continuous delivery) in mice with or without EcoHIV (HIV^+^ or HIV^−^) infection. We measured chemokine levels in the striatum (a region essential for motor and reward processing) and the hippocampus (involved in memory, learning, and other cognitive processes)—regions significantly impacted by NeuroHIV thought to contribute to HAND (Patel et al. 2017; Fitting et al. 2010, 2013; Bruce-Keller et al. 2008). Both regions harbor high levels of HIV and can display immune dysregulation and neuronal injury in response to Tat (Bruce-Keller et al. 2008; Leibrand et al. 2017). Initial data showed no differences between males and females in response to any of the treatments, so the data were collapsed across sexes. In the striatum of uninfected mice, morphine increased the concentration of several chemokines relative to saline, including CCL3, CCL4, and CCL5. In the hippocampus of uninfected mice, only the concentration of CCL4 increased following morphine exposure. Interestingly, in the uninfected mice, the pattern of changes in chemokine expression is different in several ways in response to fentanyl exposure. HIV^−^ mice exposed to fentanyl had significantly *lower* concentrations of CCL3 and higher concentrations of CCL11 and CCL17 in striatal tissue compared to those exposed to saline. In hippocampal tissue, fentanyl exposure also led to significantly lower levels of CXCL10 and higher concentrations of CCL11, unlike the response to morphine. CCL3 concentrations were unchanged relative to saline in the hippocampus of HIV^−^ mice, which is consistent with the response to morphine in this group. However, whereas both drugs led to an increase in CCL4 concentrations in both striatum and hippocampus, the increase in response to fentanyl in the hippocampus was significantly greater with morphine. To summarize, results in uninfected mice reveal differences in the effects of morphine vs fentanyl in some but not all chemokines we measured, and some of those differences were region-specific.

Among infected animals (HIV^+^), the differences in the effects of the two drugs were slightly more pronounced. Whereas morphine had no effect on CCL3 concentrations in the striatum of HIV^+^ animals, fentanyl significantly decreased CCL3 levels. Moreover, while both morphine and fentanyl significantly increased the concentrations of CCL2, CCL4, CCL5, and CCL11 in striatum, fentanyl caused significantly greater increases in CCL2 than morphine. In the hippocampus of HIV + mice, fentanyl decreased CCL3 and CXCL10 concentrations, whereas morphine did not alter CCL3 concentrations in the hippocampus. Thus, CCL3 was unaffected by morphine, but its levels were decreased by fentanyl in both brain regions.

#### Hierarchical clustering reveals treatment-specific effects on chemokine concentrations in the striatum

**Fig. 3:**
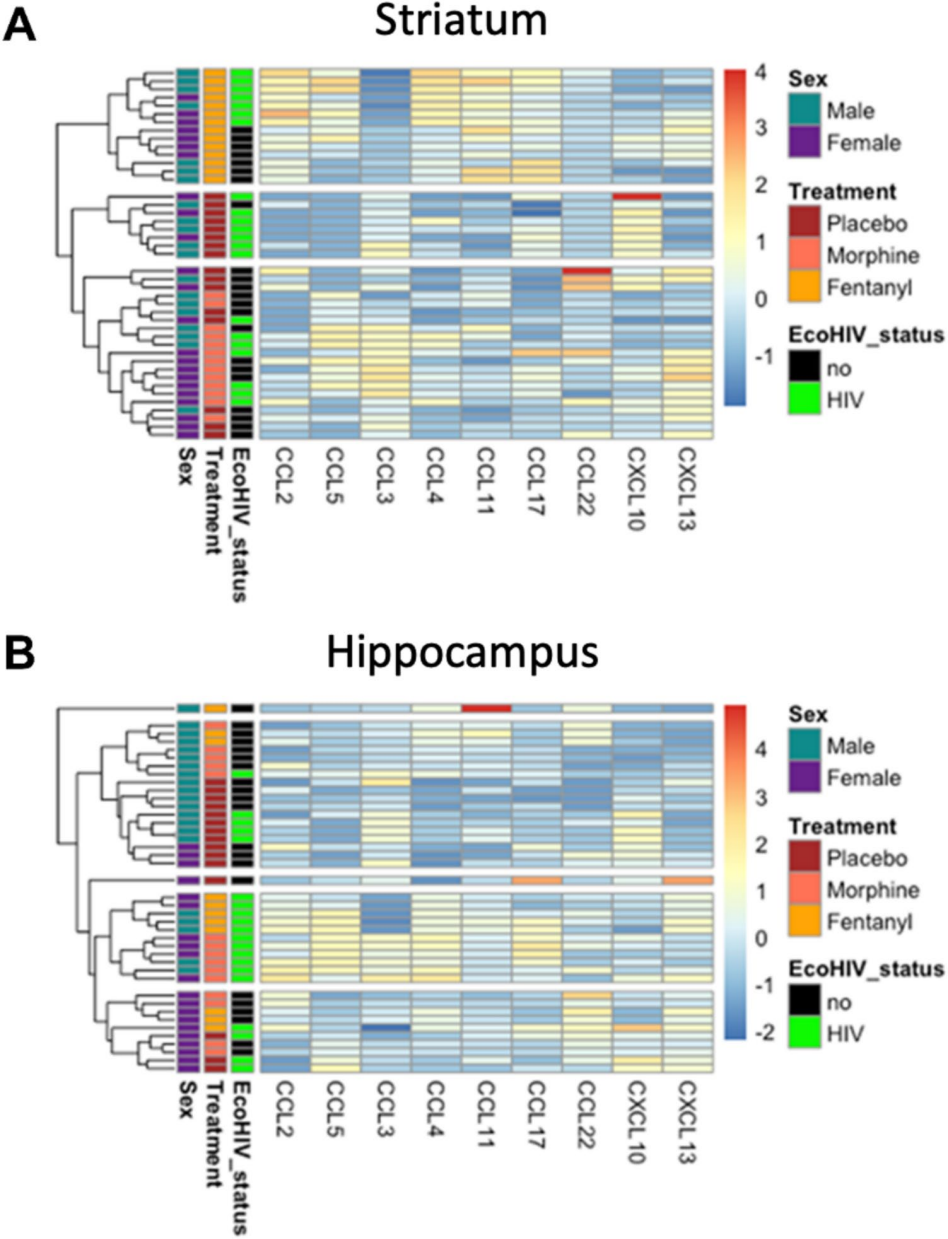
Hierarchical clustering was performed on chemokine expression levels within the striatum (**A**) (*n = *43) and hippocampus (**B**) (*n = *41). Each row represents one mouse. Relative expression levels for chemokines were visualized using a color spectrum with blue indicating lowest expression and red indicating highest expression. Brain regions with similar patterns of chemokine expression were positioned closer together. EcoHIV status, treatment exposure, and sex are labeled for each mouse. The Euclidean distance measure and complete clustering method were used for the hierarchical clustering analysis. Three distinct clusters were identified in the striatum and five clusters were identified in the hippocampus, facilitating visualization of differences between clusters

To explore the broader patterns of the chemokine response to opioids and EcoHIV, we also analyzed the data using hierarchical clustering. This method allows us to identify clusters of chemokines that exhibit similar response profiles across the different treatment groups. Analyses of the concentration changes for individual chemokines revealed drug-specific perturbations in the striatum. Hierarchical clustering analyses also showed distinct clustering of chemokines by treatment (Fig. 3a). The chemokines most influenced by drug treatment were CCL2, CCL3, CCL4, CCL5, and CCL11. Interestingly, although CCL3 was affected by both drugs, the directionality of the change was different: CCL3 decreased following fentanyl treatment but increased following morphine treatment. In the striatum, we also see clustering within the fentanyl-treated groups based on infection status: EcoHIV-infected mice treated with fentanyl had higher concentrations of CCL2, CCL4, CCL5, and CCL11 but lower levels of CCL3 and CXCL10 than the uninfected, fentanyl-treated animals.

#### Principal component analysis (PCA) reveals distinct clustering by treatment in the striatum

**Fig. 4:**
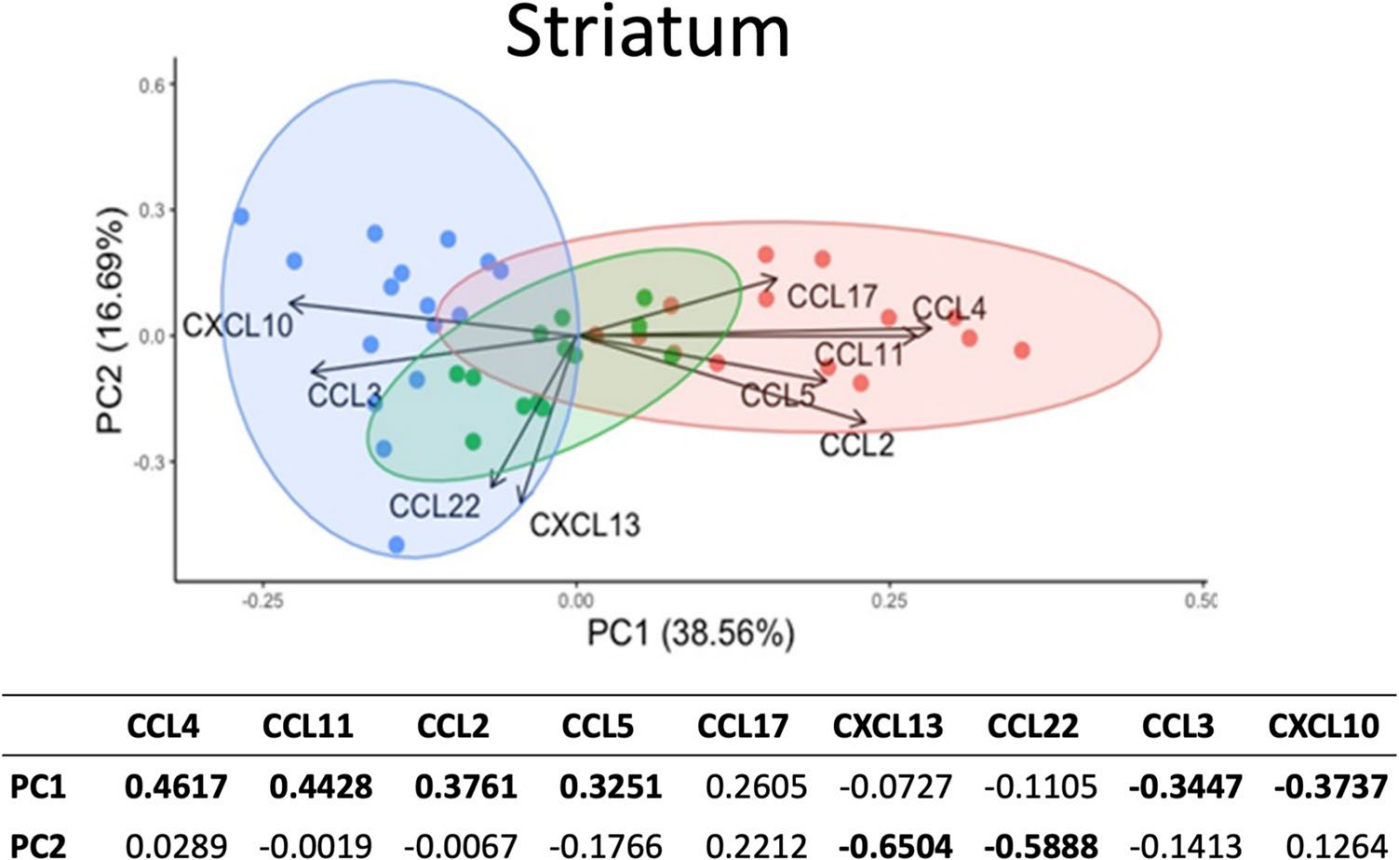
Principal component analysis of the effects of morphine and fentanyl on chemokine expression within the striatum (*n = *43). Principal component analysis was used to identify the principal components (PC1 and PC2) contributing most significantly to the variability in chemokine expression data within the striatum. Each point represents the value from one mouse and its location is influenced by its measured chemokine expression. Mice were labeled and groups were delineated based on treatment exposure with red, green, or blue, representing fentanyl, morphine, and placebo treatment, respectively. PC, principal component. The table at the bottom represents the loadings for PC1 and PC2. CCL4, CCL11, CCL2 and CCL5 have strong positive loadings for PC1 whereas CXCL10 and CCL3 have strong negative loadings for PC1. For PC2, CXCL13 and CCL22 have the strongest negative loadings

To further explore potential differences in chemokine profiles resulting from fentanyl or morphine and EcoHIV, we performed PCA on chemokine concentration data from striatal tissue. We find that the first and second components together account for 56% of the variance in chemokine concentration profile (39% to PC1 and 17% to PC2). PC1 was mainly driven by the inflammatory chemokines (in order of their relative influence): CCL4, CCL11, CCL2, CCL5 (whose loadings were the greatest positive values) and CCL3 and CXCL10 (whose loadings were the most negative values) (Fig. 4, 1S). The fact that the chemokines most influenced by fentanyl align with those contributing most to PC1 in the PCA suggests that fentanyl has a distinct impact on the concentrations of these chemokines compared to morphine or saline.

#### Hierarchical clustering of chemokines from the hippocampus reveals fewer treatment-specific effects than observed in striatum

We find a less distinct clustering pattern within the hippocampus. We see five main clusters, although two only comprise a single mouse each. In the other three, clustering around treatment group was much less clear compared to data from the striatum. There was more distinct clustering around sex than observed in the striatum; one cluster comprises all females and another is predominantly male. Additionally, one cluster comprised only EcoHIV-infected mice, which had higher levels of CCL2, CCL4, and CCL5 than the other clusters. Within this cluster of HIV + mice, half had been treated with fentanyl and the other half with morphine; as we saw in the striatum, the CCL3 level is lower in the fentanyl-treated mice compared to the morphine-treated mice in the same cluster (Fig. 3b).

#### PCA for chemokines in the hippocampus

**Fig. 5:**
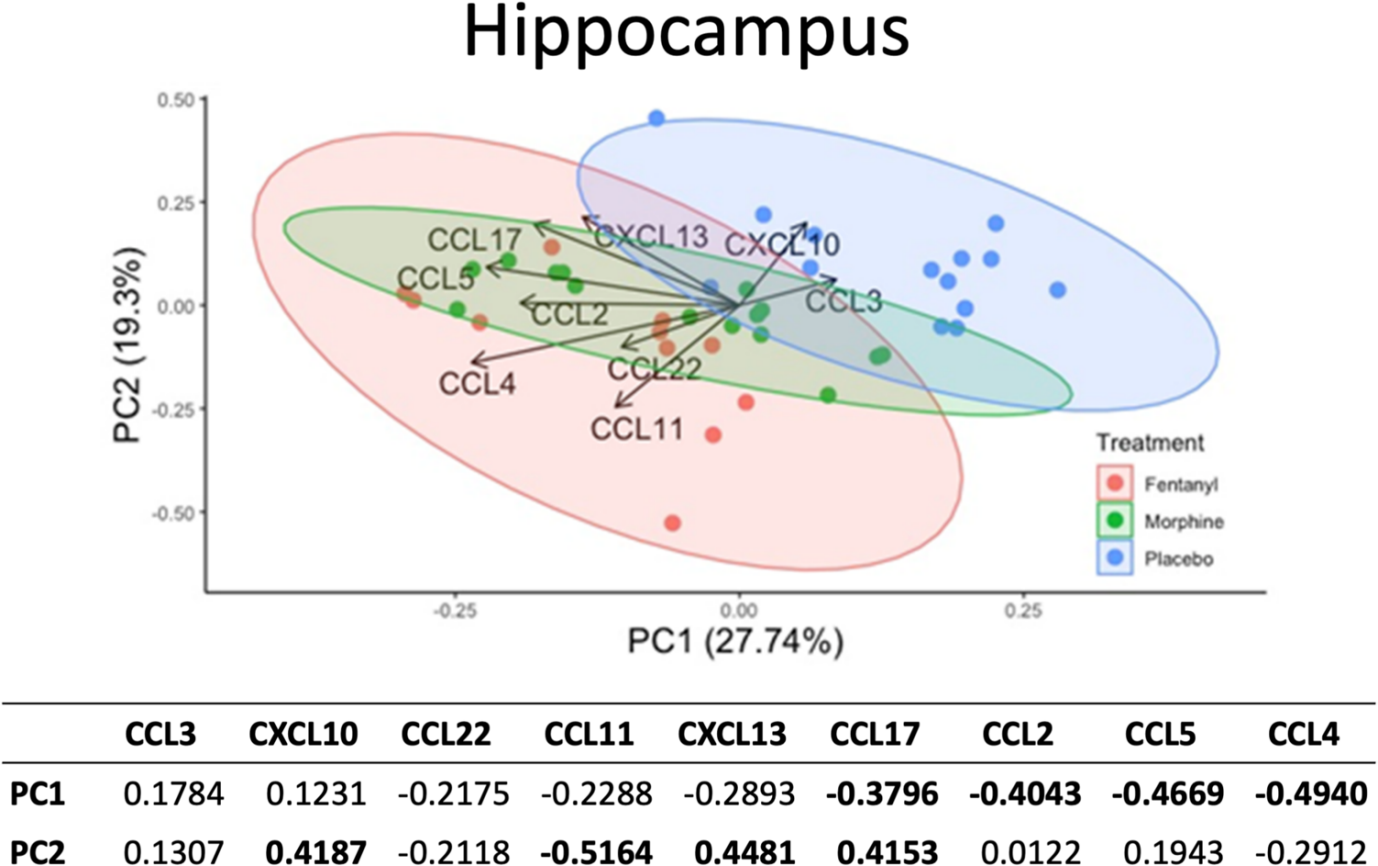
Principal component analysis of the effects of morphine and fentanyl on chemokine expression within the hippocampus (*n = *41). Principal component analysis was used to identify the principal components (PC1 and PC2) contributing most significantly to the variability in chemokine expression data within the hippocampus. Each point represents value from one mouse and its location is influenced by its measured chemokine expression. Mice were labeled and groups were delineated based on treatment exposure with red, green, or blue, representing fentanyl, morphine, and placebo treatment, respectively. PC, principal component. The table at the bottom represents the loadings for PC1 and PC2. CCL4, CCL5, CCL2, and CCL17 have strong negative loadings for PC1. CXCL14, CXCL10 and CCL17 have strong positive loadings for PC2 and CCL11 has a strong negative loading for PC2

Results of a PCA analysis of chemokine response in hippocampus by treatment group indicates that PC1 (27.7%), PC2 (19.3%), and PC3 (14.87%) account for the majority of variance (Fig. 3S). The decision to include PC3 was based on the scree plot, which showed a noticeable bend after PC3. This indicates that the first three principal components capture the most significant variation in the data. The component loadings for PC1 demonstrated that PC1 was mainly driven by (in order of influence): CCL4, CCL5, CCL2, and CCL17 (Fig. 5, 2S), which, in contrast to the striatum, had highly negative values. The contrasting loadings of key chemokines (CCL4, CCL5, CCL2, CCL17) on PC1 between the striatum and hippocampus underscore the region-specific nature of chemokine regulation within the brain.

### Evidence of differences in the effects of morphine vs fentanyl on the expression of tight junction-related proteins in mice infected with EcoHIV

**Fig. 6:**
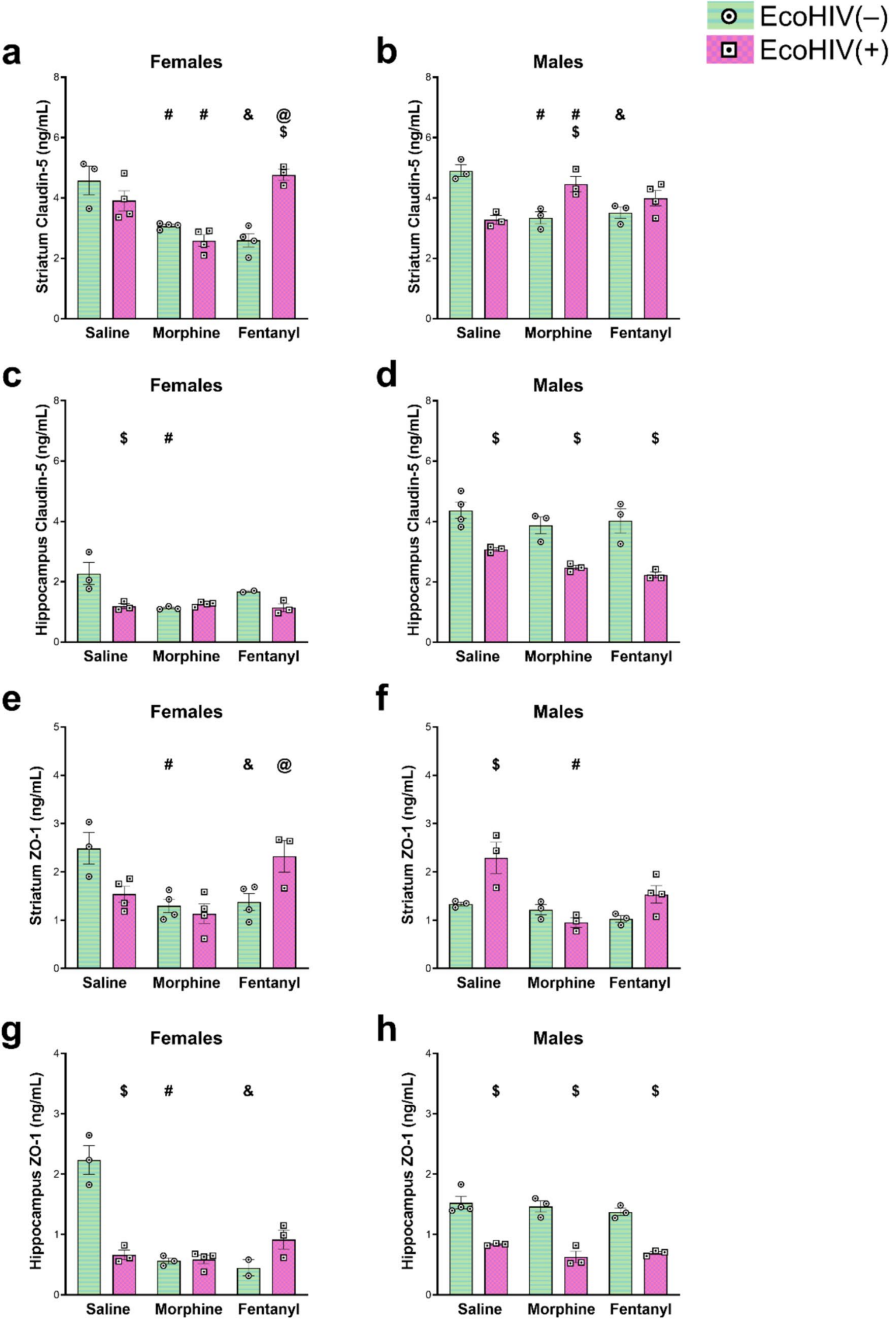
Effects of morphine, fentanyl, and EcoHIV on claudin-5 and ZO-1 levels in the striatum and hippocampus of female and male mice. EcoHIV(–) mice are depicted by horizontal green lines and white circles with a black dot. EcoHIV(+) mice are depicted by pink squares and white squares with a black dot. Data represent the mean claudin-5 and ZO-1 concentrations for females and males ± SEM, sampled from *n = *3–4 per experimental group. Sidak’s multiple comparison post-hoc analysis was used to account for multiple testing. Significant differences at α < 0.05 are denoted by ^#^*p < *0.05 saline vs. morphine, ^&^*p < *0.05 saline vs. fentanyl, ^@^*p < *0.05 morphine vs. fentanyl, or ^$^*p < *0.05 EcoHIV(–) vs. EcoHIV(+) for post-hoc testing

To investigate possible opioid-specific effects on blood–brain barrier (BBB) integrity, we tracked changes in the expression of two different tight junction-associated proteins—claudin-5 and ZO-1—following morphine or fentanyl exposure. Again, we focused on expression in the striatum and hippocampus. In this case, we saw evidence of sex-dependent effects in both HIV^−^ and HIV^+^ mice, so data were analyzed separately for males and females (Fig. 6). Statistical details, including the *F* values and degrees of freedom, are provided in Table 2.

**Table 2:** Effects of fentanyl or morphine and EcoHIV infection on claudin-5 and ZO-1 concentrations in the striatum and hippocampus

Antiretroviral Drug Concentrations	Opioid/Treatment (Main effect)		EcoHIV (Main Effect)		Interaction	
**Females**	*F*_(2,16)_	*p*	*F*_(1,16)_	*p*	*F*_(2,16)_	*p*
Claudin-5 Striatum	15.52	**0.0002**⟣	2.471	0.13	18.14	** < 0.0001**⩷
Claudin-5 Hippocampus*	5.767	**0.01**⟣	13.28	**0.003**⫚	7.391	**0.008**⩷
ZO-1 Striatum	7.945	**0.004**⟣	0.099	0.75	9.236	**0.002**⩷
ZO-1 Hippocampus*	26.24	** < 0.0001**⟣	10.74	**0.006**⫚	31.15	** < 0.0001**⩷
**Males**	F_(2,13)_	*p*	F_(1,13)_	*p*	F_(2,13)_	*p*
Claudin-5 Striatum	1.231	0.32	0.003	0.95	20.77	** < 0.0001**⩷
Claudin-5 Hippocampus	3.99	**0.04**⟣	58.59	** < 0.0001**⫚	0.6082	0.55
ZO-1 Striatum	9.493	**0.002**⟣	8.324	**0.01**⫚	6.301	**0.01**⩷
ZO-1 Hippocampus	2.226	0.14	126.6	** < 0.0001**⫚	0.59	0.56

*Female hippocampus treatment main effect and interaction [*F*_(2,12)_]; main effect for EcoHIV [*F*_(1,12)_]

Main effects are represented by significant main effect of treatment (⟣) [morphine or fentanyl vs. saline-treated], significant main effect of EcoHIV status (⫚) [EcoHIV(−) vs. EcoHIV(+)], and the interaction between treatment and EcoHIV status (⩷)

Overall, we see an effect of EcoHIV infection status alone on tight junction protein expression, particularly in the hippocampus of male mice, where claudin-5 and ZO-1 expression is significantly decreased across all treatment groups (morphine, fentanyl, and saline controls) in EcoHIV-infected mice as compared to the uninfected controls within the same treatment group.

In HIV^−^ mice, we see a decrease in claudin-5 expression in the striatum in both males and females and in response to both morphine and fentanyl; a sex- and opioid-independent effect. In HIV^+^ mice, we see a sex-specific effect of morphine on claudin-5 expression in the striatum; expression increases in males and decreases in females in HIV^+^ striatal samples. Looking at ZO-1 expression, we see an opioid-independent decrease in expression in striatal tissue from female, but not male, HIV^−^ mice. In HIV^+^ mice, effects on ZO-1 expression in striatum is both opioid- and sex-dependent. Morphine exposure leads to a decrease in ZO-1 expression in male mice, but has no effect in female mice, whereas fentanyl decreases ZO-1 in male mice and increases expression in female mice, which is also significantly greater than ZO-1 expression in the morphine treated HIV^+^ mice. There is no effect of morphine or fentanyl exposure on ZO-1 expression in hippocampal tissue from HIV^+^ mice, whereas in HIV^−^ mice, ZO-1 expression decreases following either morphine or fentanyl exposure, but only in female mice.

In summary, our tight junction protein expression data demonstrate that there are opioid-specific, sex-specific, and region-specific effects on these important BBB proteins. Furthermore, EcoHIV infection itself causes BBB impairment, which is particularly pronounced in male mice.

### Evidence of differences in the effects of morphine vs fentanyl on the concentration of antiretroviral drugs in mice infected with EcoHIV

Our prior work has shown that chronic morphine exposure inhibits the accumulation of antiretroviral drugs in brain tissue of HIV-1 Tat transgenic mice (Leibrand et al. 2019). In this study, we used the EcoHIV infection model to look for differences in the effects of morphine vs fentanyl on the concentration of three different ARV drugs—abacavir, lamivudine, and dolutegravir —in striatum and hippocampus (plasma concentration served as control) in infected and non-infected animals. Again, initial data indicated no sex-based differences, so all data from males and females were collapsed.

**Fig. 7:**
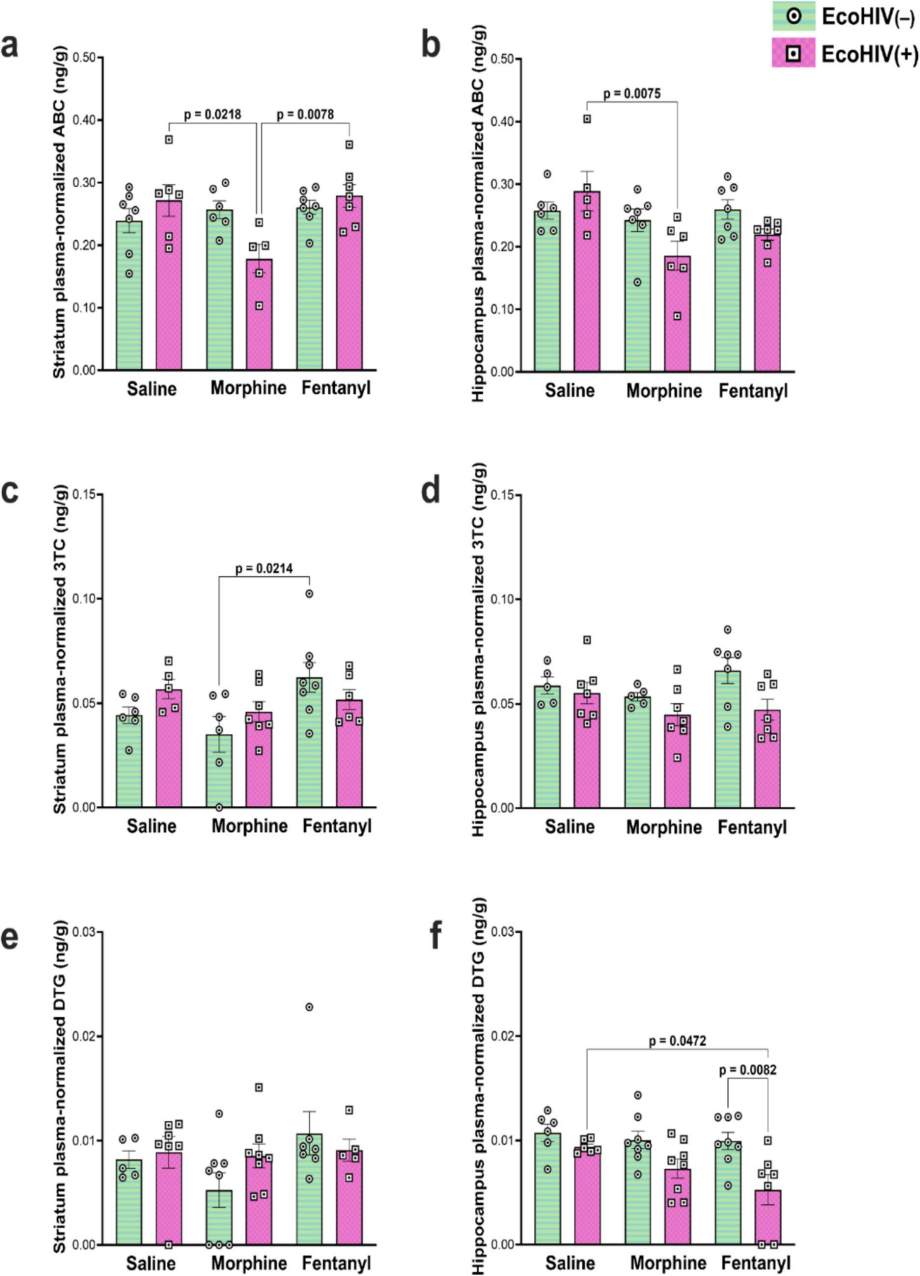
Effects of morphine, fentanyl, and EcoHIV on plasma-normalized tissue concentrations of antiretroviral drugs in the striatum and hippocampus. EcoHIV(–) mice are depicted by horizontal green lines and white circles with a black dot. EcoHIV(+) mice are depicted by pink squares and white squares with a black dot. Sidak’s multiple comparison post-hoc analysis was used to account for multiple testing. Data represent the mean plasma-normalized tissue concentration for each ARV ± SEM, sampled from *n = *5–8 per experimental group

In HIV^−^ animals, neither morphine nor fentanyl significantly altered striatal lamivudine concentrations compared to saline. However, lamivudine concentrations were significantly lower in morphine-treated animals compared to fentanyl-treated animals (*p = *0.0214). In HIV^+^ animals, morphine exposure led to a decrease in plasma-normalized abacavir concentrations in the striatum and hippocampus; there were no changes in lamivudine or dolutegravir concentrations in either the striatum or hippocampus of HIV^+^ animals following morphine exposure. In contrast, fentanyl exposure led to a decrease in plasma-normalized dolutegravir concentrations in HIV^+^ animals, but only in the hippocampus; there were no changes in abacavir or lamivudine concentrations in either the striatum or hippocampus following fentanyl exposure. These data indicate that fentanyl and morphine differentially affect ARV drug concentrations in the brains of EcoHIV-infected mice (Fig. 7). Statistical details, including the *F* values and degrees of freedom, are provided in Table 3.

**Table 3:** Effects of fentanyl or morphine and EcoHIV infection on antiretroviral concentrations in plasma and tissue

Antiretroviral Drug Concentrations	Opioid/Treatment (Main effect)		EcoHIV (Main Effect)		Interaction	
**Plasma**	*F*_(2,39)_	*p*	*F*_(1,39)_	*p*	*F*_(2,39)_	*p*
Plasma ABC	3.509	**0.03**⟣	0.8078	0.37	4.116	**0.02**⩷
Plasma 3 TC	0.2303	0.79	0.3826	0.53	2.768	0.07
Plasma DTG	0.2729	0.76	1.121	0.29	0.3755	0.68
**Striatum**	*F*_(2,32)_	*p*	*F*_(1,32)_	*p*	*F*_(2,32)_	*p*
T:P* ABC	3.947	**0.02**⟣	0.3627	0.55	4.829	**0.01**⩷
T:P* DTG	3.942	**0.02**⟣	0.4678	0.49	1.493	0.24
T:P* 3 TC	3.919	**0.03**⟣	0.7103	0.40	2.291	0.11
**Hippocampus**	*F*_(2,32)_	*p*	*F*_(1,32)_	*p*	*F*_(2,32)_	*p*
T:P* ABC	4.912	**0.01**⟣	2.114	0.15	2.931	0.06
T:P* DTG	4.719	**0.01**⟣	13.57	**0.0008**⫚	1.786	0.18
T:P* 3 TC	1.396	0.26	5.905	**0.02**⫚	1.178	0.32

*T:P: plasma-normalized tissue concentrations

*ABC* abacavir, *DTG* dolutegravir, *3 TC* lamivudine

Main effects are represented by significant main effect of treatment (⟣) [morphine or fentanyl vs. saline-treated], significant main effect of EcoHIV status (⫚) [EcoHIV(−) vs. EcoHIV(+)], and the interaction between Treatment and EcoHIV status (⩷)

## Discussion

Our study is among the first to investigate the effects of fentanyl within the context of HIV infection. It is also the first to directly compare fentanyl to other commonly used opioids to explore drug-specific differences in their impact on HIV pathology in the brain. Fentanyl and its analogs act fundamentally differently from the prototypical opioid morphine, as well as other opioids such as heroin and oxycodone. Fentanyl (and its analogues) have unique pharmacologic properties at MOR and non-opioid receptors that differ dramatically from other opioids (Vardanyan and Hruby 2014; Sutcliffe et al. 2022; Kelly et al. 2023; Torralva et al. 2020; Carranza-Aguilar et al. 2022; Yarotskyy et al. 2024). Administration of fentanyl can result in biphasic and dose-dependent changes in brain oxygenation and temperature, which can result in transient hypoxia and increased thermal stress (Kiyatkin and Choi 2024; Choi et al. 2024). Additionally, chronic fentanyl is reported to increase apoptosis, oxidative stress, neuroinflammation and disrupt *N*-methyl-D-aspartate subunit and dopamine receptor levels (Alzu’bi et al. 2024). The possibility that fentanyl uniquely interacts with HIV pathology to worsen clinical outcomes in patients with comorbid HIV and OUD is likely to have significant implications for the development of treatment strategies to benefit PLWH.

### Fentanyl-specific effects on chemokine signaling in the striatum of uninfected mice

We find that the effects of 5 days of fentanyl exposure were distinct from morphine exposure in several ways. Most pronounced were the differences in chemokine expression levels. We tracked changes in the expression of 13 different chemokines, many of which were chosen for their roles in inflammation, immune regulation, and disease pathology. In past studies from our group and others, several of these, including CCL2, CCL3, CCL4, CCL5, CCL17, CCL20, CCL22, CXCL9, CXCL10, and CXCL13 were affected by opioid exposure (Rademeyer et al. 2024; Gonek et al. 2018; Avdoshina et al. 2011; Davis et al. 2007). For example, in a HIV-1 Tat transgenic mouse model (Rademeyer et al. 2024), we have previously reported that fentanyl exposure caused pronounced increases CCL4, CCL20 (within the striatum), CXCL10, and CXCL13, but decreases CCL3, CCL17, CCL20 (in the hippocampus) and CXCL9. Although chemokine levels were also dysregulated following fentanyl exposure in EcoHIV-infected mice, not unexpectedly, the extent and pattern of expression differed between Tat and EcoHIV models.

Results of our hierarchical clustering and PCA analyses largely align with each other and support the key finding that there is a fentanyl-specific effect on chemokine signaling patterns that is independent of infection status. From these analyses, we can more precisely define the distinctions and variability within each treatment group and by brain region (striatum vs hippocampus). In striatum, there was a clear difference in the pattern of effects of morphine vs fentanyl. The chemokines primarily driving this effect are CCL4, CCL11, CCL2, CCL5 and CCL3. The altered chemokine profile induced by fentanyl suggests a complex and nuanced immunomodulatory effect, highlighting its potential to uniquely alter neuroimmune responses. Thus, at a practical level, our data suggest there may be a profoundly dysregulated inflammatory response within the brain associated with sustained fentanyl exposure compared to morphine exposure. This difference may leave individuals who abuse fentanyl particularly susceptible to additional CNS insults such as neuroHIV. However, we acknowledge that while the drug doses were based on human doses which were allometrically scaled to the mouse model, this approach may not ensure equipotent dosing, and caution must be taken not to overinterpret the findings. Further work is needed to understand the mechanisms that drive the differential neuroimmune responses to fentanyl and morphine, as well as to gain a greater understanding of the broader consequences of fentanyl and its analogs vs ‘typical’ opioids such as heroin, morphine, and oxycodone in neuroHIV. This is especially important since the misuse of fentanyl has largely replaced heroin and other semi-synthetic opioids such as oxycodone in OUD (Ickowicz et al. 2022; Spencer et al. 2023; Larnder et al. 2022).

### The potential for interactions between fentanyl and HIV to impact chemokine expression

Within the cluster of data from the striatum of fentanyl-treated animals, there is a clear dependence on infection status. EcoHIV-infected mice had higher CCL2, CCL4, CCL5 and lower CCL3 and CXCL10 compared to uninfected mice. This suggests that HIV infection influences the immune response to fentanyl treatment, resulting in distinct immunomodulatory effects that could enhance immune cell recruitment like monocyte and macrophages. These changes could also mean a reduction in Th1-mediated immunity, as suggested by the decrease in CXCL10, which would lead to a decreased antiviral response (Gnanadurai and Fu 2016; Asensio et al. 2001). The implications for PLWH are that fentanyl may simultaneously exaggerate some immune responses, leading to more neuroinflammation, while suppressing innate antiviral activity, which may allow for increased viral replication. This combination would likely worsen clinical outcomes, particularly in terms of neuroHIV-related conditions.

Within the hippocampus, there also was a distinction in the clustering patterns of chemokines between fentanyl and morphine. However, the effects in the hippocampus are less clearly defined than in the striatum. This suggests that the hippocampus has a more variable chemokine response to opioids and to HIV exposure. Such differences may reflect distinct cellular populations, signaling pathways, or microenvironmental factors operating in these brain regions. Further elucidating the molecular mechanisms underlying these disparities is crucial for understanding the complex interplay between neuroimmune signaling and brain region-specific functions. Regardless, there was a chemokine clustering difference between fentanyl and morphine among HIV^+^ animals, further supporting the hypothesis that fentanyl’s effects are distinct from other opioids in many ways. Overall, our data from both striatum and hippocampus suggest that HIV plus fentanyl exposure contributes to neuroinflammation and neurotoxicity in both areas, but the striatum's distinct clustering suggests heightened vulnerability to these effects. Thus, we might expect fentanyl to trigger more severe impairments in cognitive functions such as decision-making, attention, and executive function in PLWH. This is consistent with the literature describing worsened HIV neuropathology with opioid use/misuse. Studies describe increased cognitive impairment and neuropsychiatric symptoms among PLWH who use opioids like heroin or fentanyl compared to people without HIV (Hauser et al. 2012; Murphy et al. 2019; Nash et al. 2019; Tamargo et al. 2021). However, there are no clinical studies to date delineating the impact of fentanyl separate from other opioids. Our data suggest such studies are warranted.

### There are sex and brain regional differences in tight junction protein expression in response to opioids and HIV infection

The EcoHIV mouse model allows us to explore the interaction between sustained opioid exposure and pathological changes in the brain downstream of HIV infection. Unlike previous studies using the Tat transgenic model, the EcoHIV model recapitulates the innate immune response to infection, which in turn influences neuroinflammation and neuropathology, including effects on the BBB. In uninfected mice, we observed decreases in ZO-1 and claudin-5 with fentanyl and with morphine exposure. However, in HIV^+^ mice, only morphine disrupted these tight junction proteins. We and others have previously reported morphine disruption of the BBB in Tat transgenic mice in in vitro studies and we have also previously reported fentanyl-mediated BBB disruption in Tat mice (Leibrand et al. 2019; Mahajan et al. 2002; Rademeyer et al. 2024). Interestingly, the tight junction changes from fentanyl exposure in this study were more modest than those reported in prior studies using Tat mice. Fentanyl-treated Tat− and HIV^−^ female mice exhibited significant decreases in claudin-5 and ZO-1 expression in the striatum compared to saline-treated counterparts. However, in HIV^+^/fentanyl-treated mice, ZO-1 showed no significant changes, and claudin-5 expression increased, suggesting a potential modulatory effect from HIV infection. The observed differences in response may be attributed to fundamental differences between the models. While the Tat transgenic model primarily affects the CNS, EcoHIV infection is systemic, leading to different dynamics in infection and immune response. Future studies should further investigate the underlying mechanisms and the roles of other immune mediators in this context (Bruce-Keller et al. 2008).

Interestingly, the current study also adds a more nuanced picture of regional and sex-dependent effects to HIV infection. Most notably, male hippocampal tissue was more susceptible to HIV-mediated decreases in claudin-5 and ZO-1 expression than in the striatum. Additionally, tight junction protein expression was not influenced by HIV in either region of female mice, supporting other reports of BBB resiliency within females following mild blast traumatic brain injury (Hubbard et al. 2022). Further, this BBB resiliency to inflammatory signals, which follow HIV infection, may be estrogen-mediated (Maggioli et al. 2016). Extending this idea to humans, clinical studies report that women with HIV show greater neurocognitive impairment than men with HIV (reviewed in Rubin and Maki 2021). This discrepancy may be because of some of the psychosocial factors influencing cognitive vulnerability, such as women having higher rates of poverty, lower literacy levels, higher rates of some mental health illnesses, and poorer access to health care, which may be the overriding factors (Rubin and Maki 2021).

### Evidence for distinct effects of fentanyl on ARV accumulation in the brains of HIV-infected mice

The potential for 5-day opioid exposure to impact ARV accumulation in the brain, particularly in regions known to be susceptible to HIV pathology, has clear implications for treatment efficacy and long-term clinical outcomes among PLWH and comorbid OUD. Our data suggest that knowing which opioid a patient has been exposed to may be important for therapeutic management. We find evidence that morphine and fentanyl differ in terms of which ARV drug is affected and which region of the brain those effects are seen (striatum vs hippocampus). In EcoHIV-infected mice (only), fentanyl exposure led to a decrease in dolutegravir concentrations in the hippocampus, whereas morphine exposure led to a decrease in abacavir concentrations in the striatum. We have previously demonstrated region-specific morphine-mediated decreases in ARV concentrations in brain tissue of Tat transgenic mice, but in that case the changes were in both Tat^+^ and Tat^−^ mice (Leibrand et al. 2019). This discrepancy likely reflects some of the fundamental differences between the models. Based on what we know about HIV pathology and the effect of ARV drugs in these regions, our current data suggest that fentanyl and its analogues are likely to affect the progression of neuroHIV differentially from heroin and morphine. Thus, PLWH taking the ARV regimen of dolutegravir/abacavir/lamivudine who are also using fentanyl might experience more heightened pathophysiological changes and potentially a greater degree of neurocognitive impairment. Since fentanyl and its analogues have largely replaced heroin and conventional opioids in OUD, more work is needed to better understand the unique interactive consequences of fentanyl on pharmacokinetics and efficacy of ARV therapies in the CNS and to gain a greater understanding of the broader consequences of fentanyl and its analogs in neuroHIV.

## Supplementary Information

Below is the link to the electronic supplementary material.

Supplementary file1 (DOCX 719 KB)

## Data Availability

Data will be made available upon request.
