# Supplementary material for: Differential effects of fentanyl compared to morphine on neuroinflammatory signaling in the brain in EcoHIV-infected mice
Source: J Neurovirol. 2025 May 30;31(3):242–61. doi: 10.1007/s13365-025-01252-z (PMC12356723; doi:10.1007/s13365-025-01252-z)
Supplement: Supplementary file 1 — Supplementary file1 (DOCX 719 KB) [file 13365_2025_1252_MOESM1_ESM.docx]

**Supplemental Information**

**Table S1.**  Post-hoc effects of saline and morphine on chemokine levels in the striatum and hippocampus.

| **Brain Region** | **Chemokine** | **Saline vs. Morphine**  **(Post-hoc effect)** | ***Adjusted p-value*** |
| --- | --- | --- | --- |
| **Striatum** | | | |
|  | [CCL2](#figure4) | Eco(+) saline < Eco(+) morphine | 0.0047 |
|  | CCL3 | Eco(–) saline < Eco(–) morphine | 0.0069 |
|  | CCL4 | Eco(–) saline < Eco(–) morphine  Eco(+) saline < Eco(+) morphine | 0.0423  < 0.0001 |
|  | CCL5 | Eco(–) saline < Eco(–) morphine  Eco(+) saline < Eco(+) morphine | 0.0009  0.0066 |
|  | CCL11 | Eco(+) saline < Eco(+) morphine | 0.0447 |
|  | CXCL10 | Eco(+) saline > Eco(+) morphine | < 0.0001 |
|  | CXCL13 | Eco(+) saline < Eco(+) morphine | 0.0131 |
| **Hippocampus** | | | |
|  | CCL2 | Eco(+) saline < Eco(+) morphine | 0.0002 |
|  | CCL4 | Eco(–) saline < Eco(–) morphine  Eco(+) saline < Eco(+) morphine | < 0.0001  < 0.0001 |
|  | CCL5 | Eco(+) saline < Eco(+) morphine | 0.0118 |
|  | CCL17 | Eco(+) saline < Eco(+) morphine | 0.0002 |
|  | CXCL10 | Eco(+) saline > Eco(+) morphine | 0.0021 |
| *Eco(–): uninfected | | |  |

Results from Šídák's post-hoc analysis in the striatum and hippocampus between treatment [morphine and saline-treated] including EcoHIV infection status.

**Table S2.**  Post-hoc effects of saline and fentanyl on chemokine levels in the striatum and hippocampus.

| **Brain Region** | **Chemokine** | **Saline vs. Fentanyl**  **(Post-hoc effect)** | ***Adjusted p-value*** |
| --- | --- | --- | --- |
| **Striatum** | | | |
|  | [CCL2](#figure4) | Eco(+) saline < Eco(+) fentanyl | <0.0001 |
|  | CCL3 | Eco(–) saline > Eco(–) fentanyl  Eco(+) saline > Eco(+) fentanyl | 0.0338  < 0.0001 |
|  | CCL4 | Eco(–) saline < Eco(–) fentanyl  Eco(+) saline < Eco(+) fentanyl | 0.0007  < 0.0001 |
|  | CCL5 | Eco(+) saline < Eco(+) fentanyl | 0.0001 |
|  | CCL11 | Eco(–) saline < Eco(–) fentanyl  Eco(+) saline < Eco(+) fentanyl | 0.0004  < 0.0001 |
|  | CCL17 | Eco(–) saline < Eco(–) fentanyl | < 0.0001 |
|  | CXCL10 | Eco(+) saline > Eco(+) fentanyl | < 0.0001 |
| **Hippocampus** | | | |
|  | CCL2 | Eco(+) saline < Eco(+) fentanyl | 0.0019 |
|  | CCL3 | Eco(+) saline > Eco(+) fentanyl | < 0.0001 |
|  | CCL4 | Eco(–) saline < Eco(–) fentanyl  Eco(+) saline < Eco(+) fentanyl | < 0.0001  < 0.0001 |
|  | CCL11 | Eco(–) saline < Eco(–) fentanyl | 0.0176 |
|  | CCL22 | Eco(–) saline < Eco(–) fentanyl | 0.0029 |
|  | CXCL10 | Eco(–) saline > Eco(–) fentanyl  Eco(+) saline > Eco(+) fentanyl | 0.0073  < 0.0001 |
| *Eco(–): uninfected | | |  |

Results from Šídák's post-hoc analysis in the striatum and hippocampus between treatment [fentanyl and saline-treated] including EcoHIV infection status.

**Table S3.**  Post-hoc effects of morphine and fentanyl on chemokine levels in the striatum and hippocampus.

| **Brain Region** | **Chemokine** | **Morphine vs. Fentanyl**  **(Post-hoc effect)** | ***Adjusted p-value*** |
| --- | --- | --- | --- |
| **Striatum** | | | |
|  | [CCL2](#figure4) | Eco(+) morphine < Eco(+) fentanyl | 0.0238 |
|  | CCL3 | Eco(–) morphine > Eco(–) fentanyl  Eco(+) morphine > Eco(+) fentanyl | < 0.0001  < 0.0001 |
|  | CCL11 | Eco(–) morphine < Eco(–) fentanyl | 0.0118 |
|  | CCL17 | Eco(–) morphine < Eco(–) fentanyl | 0.0108 |
| **Hippocampus** | | | |
|  | CCL3 | Eco(+) morphine > Eco(+) fentanyl | < 0.0001 |
|  | CCL4 | Eco(–) morphine < Eco(–) fentanyl | < 0.0001 |
| *Eco(–): uninfected group | | |  |

Results from Šídák's post-hoc analysis in the striatum and hippocampus between treatment [morphine and fentanyl] including EcoHIV infection status.

**Table S4.**  Post-hoc effects of EcoHIV infection on chemokine levels in the striatum and hippocampus.

| **Brain Region** | **Chemokine** | **Eco(–) vs. Eco(+)**  **(Post-hoc effect)** | ***Adjusted p-value*** |
| --- | --- | --- | --- |
| **Striatum** | | | |
|  | [CCL2](#figure4) | Eco(–) fentanyl < Eco(+) fentanyl | 0.0249 |
|  | CCL3 | Eco(–) fentanyl > Eco(+) fentanyl | 0.0333 |
|  | CCL4 | Eco(–) morphine < Eco(+) morphine  Eco(–) fentanyl < Eco(+) fentanyl | 0.002  < 0.0001 |
|  | CCL5 | Eco(–) fentanyl < Eco(+) fentanyl | 0.0457 |
|  | CCL17 | Eco(–) saline < Eco(+) saline | 0.0123 |
|  | CCL22 | Eco(–) saline > Eco(+) saline | 0.0316 |
|  | CXCL10 | Eco(–) saline < Eco(+) saline | 0.0155 |
|  | CXCL13 | Eco(–) saline > Eco(+) saline | 0.0009 |
| **Hippocampus** | | | |
|  | CCL2 | Eco(–) morphine < Eco(+) morphine | 0.0059 |
|  | CCL3 | Eco(–) morphine < Eco(+) morphine  Eco(–) fentanyl > Eco(+) fentanyl | 0.0008  0.0004 |
|  | CCL4 | Eco(–) saline < Eco(+) saline  Eco(–) morphine < Eco(+) morphine | < 0.0001  < 0.0001 |
|  | CCL5 | Eco(–) morphine < Eco(+) morphine | 0.0013 |
|  | CCL17 | Eco(–) morphine < Eco(+) morphine  Eco(–) fentanyl < Eco(+) fentanyl | 0.0005  0.0259 |
|  | CXCL10 | Eco(–) saline < Eco(+) saline | 0.0149 |
| *Eco(–): uninfected group | | |  |

Results from Šídák's post-hoc analysis in the striatum and hippocampus between EcoHIV infection status.

**Table S5.**  Post-hoc effects of morphine, fentanyl and EcoHIV infection on antiretroviral concentrations in the striatum and hippocampus.

| **Brain Region** (Antiretroviral) | Multiple Comparison | Adjusted p-value |
| --- | --- | --- |
| **Striatum** | | |
| Abacavir | Eco(+) saline > Eco(+) morphine  Eco(+) morphine < Eco(+) fentanyl | 0.0218  0.0078 |
| Lamivudine | Eco(–) morphine < Eco(–) fentanyl | 0.0214 |
| **Hippocampus** | | |
| Abacavir | Eco(+) saline < Eco(+) morphine | 0.0075 |
| Dolutegravir | Eco(+) saline > Eco(+) fentanyl  Eco(–) fentanyl > Eco(+) fentanyl | 0.0472  0.0082 |
| *Eco(–): uninfected group; Results from Šídák's post-hoc analysis in the striatum and hippocampus between EcoHIV infection status. | | |

**Table S6.**  Post-hoc effects of morphine, fentanyl and EcoHIV infection on claudin-5 and ZO-1 concentrations in the striatum and hippocampus

| Tight Junction (**Brain Region**) | Multiple comparison | Adjusted p-value |
| --- | --- | --- |
| **Females** | | |
| Claudin-5 (**Striatum**) | Eco(–) saline > Eco(–) morphine  Eco(+) saline > Eco(+) morphine  Eco(–) saline > Eco(–) fentanyl  Eco(+) morphine < Eco(+) fentanyl  Eco(–) fentanyl < Eco(+) fentanyl | 0.0094  0.0144  0.0007  0.0003  0.0003 |
| Claudin-5 (**Hippocampus**) | Eco(–) saline > Eco(–) morphine  Eco(–) saline > Eco(+) saline | 0.0031  0.0048 |
| ZO-1 (**Striatum**) | Eco(–) saline > Eco(–) morphine  Eco(–) saline > Eco(–) fentanyl  Eco(+) morphine < Eco(+) fentanyl | 0.0142  0.0245  0.0142 |
| ZO-1 (**Hippocampus**) | Eco(–) saline > Eco(–) morphine  Eco(–) saline > Eco(–) fentanyl  Eco(–) saline > Eco(+) saline | <0.0001  <0.0001  <0.0001 |
| **Males** | | |
| Claudin-5 (**Striatum**) | Eco(–) saline > Eco(–) morphine  Eco(+) saline < Eco(+) morphine  Eco(–) saline > Eco(–) fentanyl  Eco(–) saline > Eco(+) saline  Eco(–) morphine > Eco(+) morphine | 0.0025  0.0218  0.0064  0.0017  0.0330 |
| Claudin-5 (**Hippocampus**) | Eco(–) saline > Eco(+) saline  Eco(–) morphine > Eco(+) morphine  Eco(–) fentanyl < Eco(+) fentanyl | 0.0136  0.0124  0.0016 |
| ZO-1 (**Striatum**) | Eco(+) saline < Eco(+) morphine  Eco(–) saline > Eco(+) saline | 0.0010  0.0166 |
| ZO-1 (**Hippocampus**) | Eco(–) saline > Eco(+) saline  Eco(–) morphine > Eco(+) morphine  Eco(–) fentanyl < Eco(+) fentanyl | 0.0002  <0.0001  0.0005 |
| *Eco(–): uninfected group; Results from Šídák's post-hoc analysis in the striatum and hippocampus between EcoHIV infection status in males and females. | | |
|  | | |

**Figure 1S.** Heat map of PCA loadings within the striatum


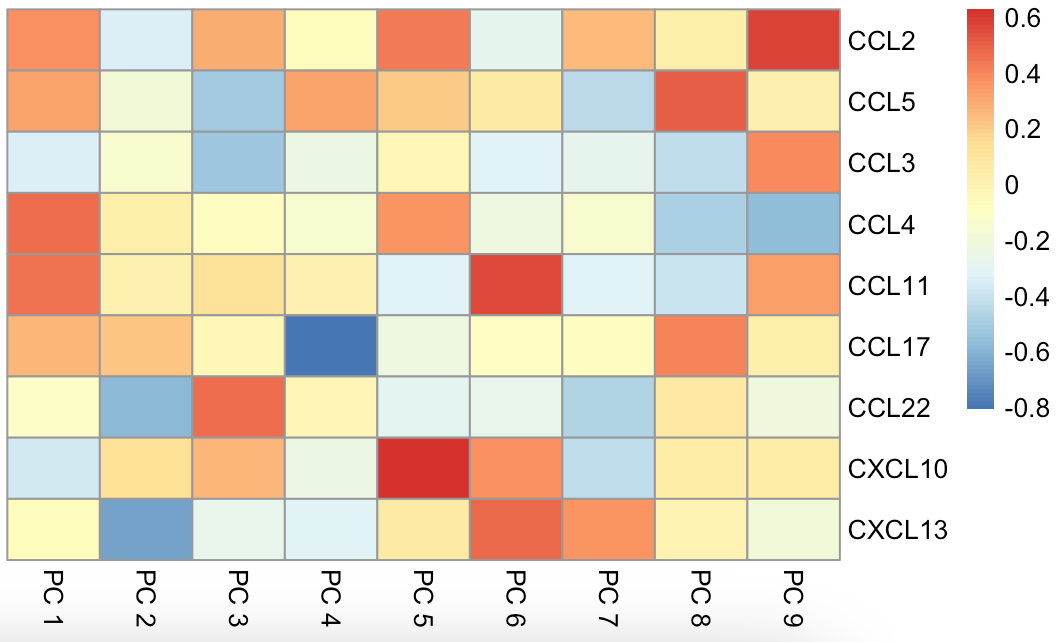


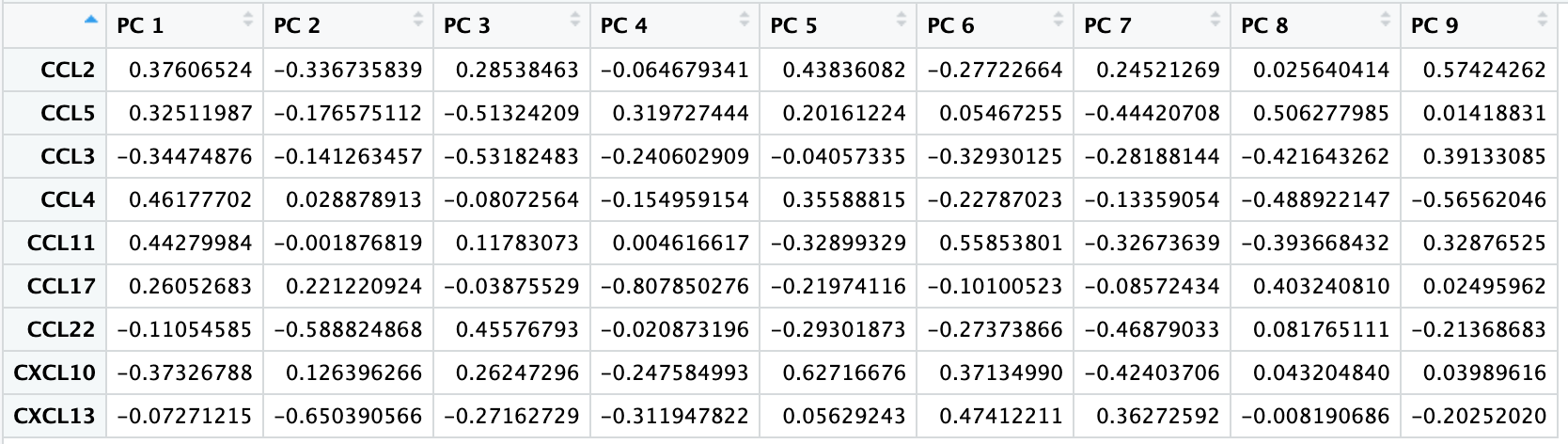
Loadings versus PC values for PCA in the striatum

**Figure 2S**. Heat map of PCA loadings within the hippocampus


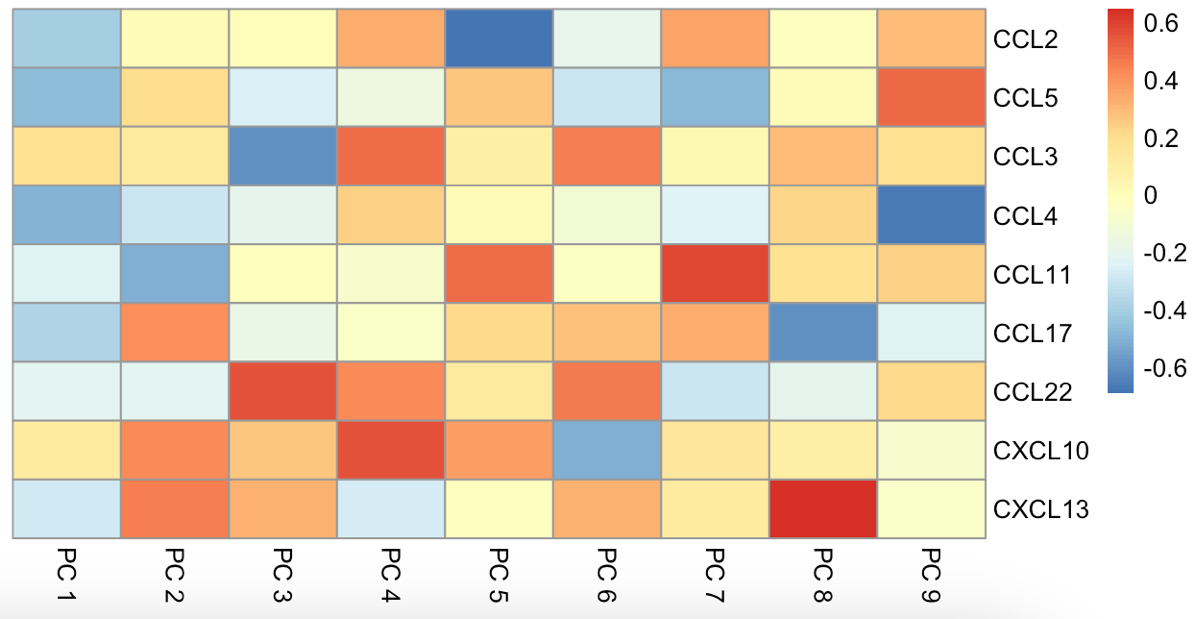


Loadings versus PC values for PCA in the striatum


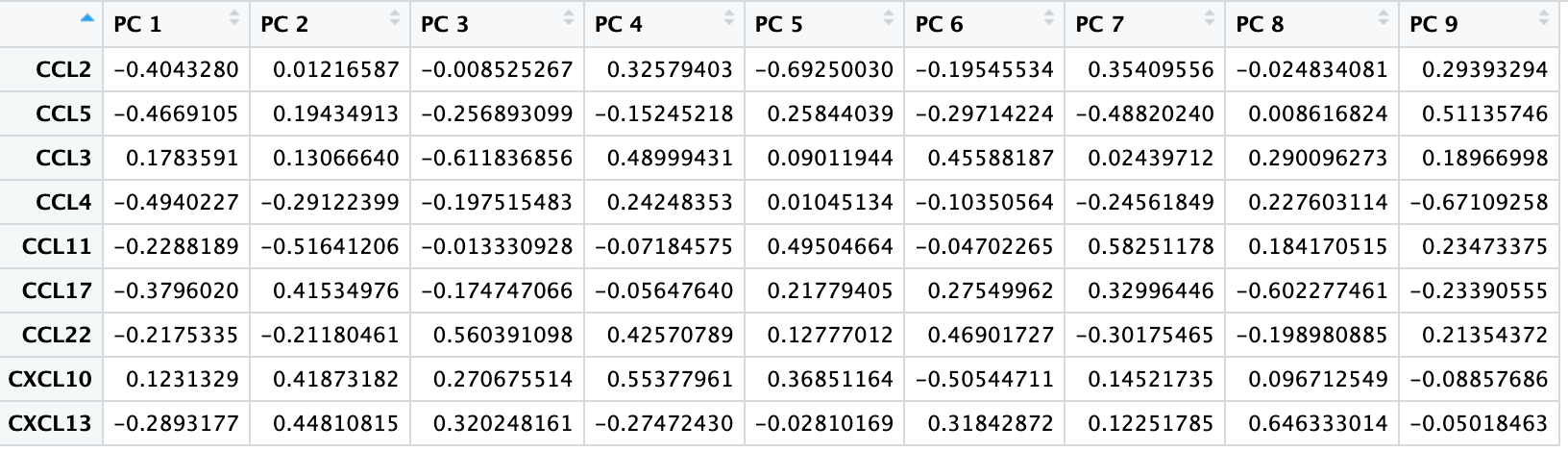


**Figure 3S.** PCA plot of hippocampus tissue chemokine expression principal component analysis labeled by opioid exposure plotted by PC1 and PC3 (A) and PC2 and PC3 (B).


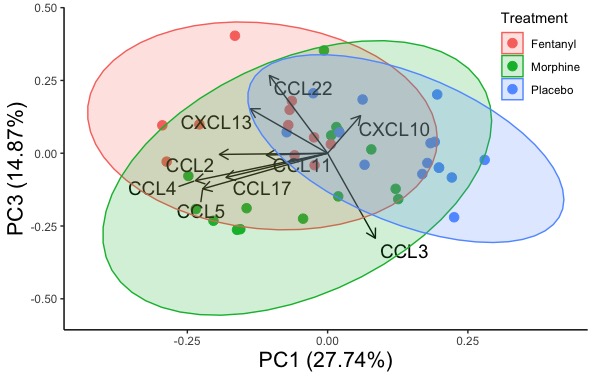


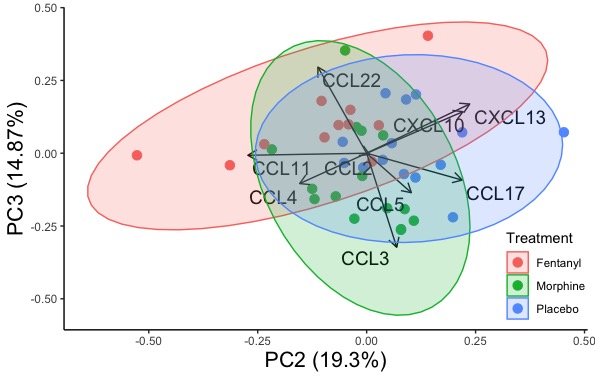


**Figure 3S.** Chemokine expression within the striatum (n= 43) and hippocampus (n= 41) were measured. Although PC1 and PC2 represented most of the variability within the hippocampus chemokine expression data, PC3 represented 14.87% of variability and was before the elbow point on the eigenvalue scree plot. Each dot represents data from one mouse and its location is influenced by its measured chemokine expression. Mice were labeled and groups were delineated based on treatment exposure with red, green, or blue, representing fentanyl, morphine, and placebo treatment, respectively. PC, principal component.

## **
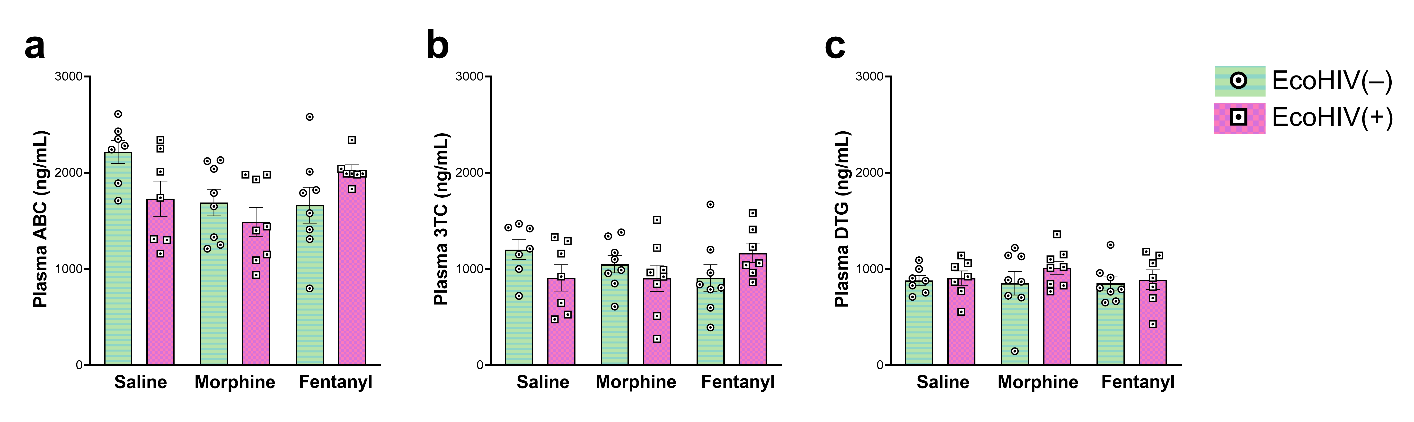
 Fig 4S.** Plasma concentrations of abacavir, lamivudine, and dolutegravir are not influenced by morphine, fentanyl or EcoHIV status. EcoHIV(–) mice are depicted by horizontal green lines and white circles with a black dot. EcoHIV(+) mice are depicted by pink squares and white squares with a black dot. Data represents the mean plasma concentration for each ARV ± SEM, sampled *from* *n* = 7 -8 per experimental group.
